# Supplementary material for: Iterative improvement in the automatic modular design of robot swarms
Source: PeerJ Comput Sci. 2020 Dec 7;6:e322. doi: 10.7717/peerj-cs.322 (PMC7924708; doi:10.7717/peerj-cs.322)
Supplement: Supplemental Information 3 [file peerj-cs-06-322-s003.zip › argos3/doc/api/standalone/a00420_source.html]

ARGoS: plugins/robots/eye-bot/control\_interface/ci\_eyebot\_light\_sensor.cpp Source File


- Main Page
- Related Pages
- Namespaces
- Classes
- Files

- File List
- File Members

# plugins/robots/eye-bot/control\_interface/ci\_eyebot\_light\_sensor.cpp

Go to the documentation of this file.

```
00001 
00007 #include "ci_eyebot_light_sensor.h"
00008 
00009 #ifdef ARGOS_WITH_LUA
00010 #include <argos3/core/wrappers/lua/lua_utility.h>
00011 #endif
00012 
00013 namespace argos {
00014 
00015    /****************************************/
00016    /****************************************/
00017 
00018    static CRadians SPACING = CRadians(ARGOS_PI / 12.0f);
00019    static CRadians START_ANGLE = SPACING * 0.5f;
00020 
00021    /****************************************/
00022    /****************************************/
00023 
00024    CCI_EyeBotLightSensor::CCI_EyeBotLightSensor() :
00025       m_tReadings(24) {
00026       for(size_t i = 0; i < 24; ++i) {
00027          m_tReadings[i].Angle = START_ANGLE + i * SPACING;
00028          m_tReadings[i].Angle.SignedNormalize();
00029       }
00030    }
00031 
00032    /****************************************/
00033    /****************************************/
00034 
00035    const CCI_EyeBotLightSensor::TReadings& CCI_EyeBotLightSensor::GetReadings() const {
00036      return m_tReadings;
00037    }
00038 
00039    /****************************************/
00040    /****************************************/
00041 
00042 #ifdef ARGOS_WITH_LUA
00043    void CCI_EyeBotLightSensor::CreateLuaState(lua_State* pt_lua_state) {
00044       CLuaUtility::OpenRobotStateTable(pt_lua_state, "light");
00045       for(size_t i = 0; i < GetReadings().size(); ++i) {
00046          CLuaUtility::StartTable(pt_lua_state, i+1                           );
00047          CLuaUtility::AddToTable(pt_lua_state, "angle",  m_tReadings[i].Angle);
00048          CLuaUtility::AddToTable(pt_lua_state, "value",  m_tReadings[i].Value);
00049          CLuaUtility::EndTable  (pt_lua_state                                );
00050       }
00051       CLuaUtility::CloseRobotStateTable(pt_lua_state);
00052    }
00053 #endif
00054 
00055    /****************************************/
00056    /****************************************/
00057 
00058 #ifdef ARGOS_WITH_LUA
00059    void CCI_EyeBotLightSensor::ReadingsToLuaState(lua_State* pt_lua_state) {
00060       lua_getfield(pt_lua_state, -1, "light");
00061       for(size_t i = 0; i < GetReadings().size(); ++i) {
00062          lua_pushnumber(pt_lua_state, i+1                 );
00063          lua_gettable  (pt_lua_state, -2                  );
00064          lua_pushnumber(pt_lua_state, m_tReadings[i].Value);
00065          lua_setfield  (pt_lua_state, -2, "value"         );
00066          lua_pop(pt_lua_state, 1);
00067       }
00068       lua_pop(pt_lua_state, 1);
00069    }
00070 #endif
00071 
00072 
00073    /****************************************/
00074    /****************************************/
00075 
00076    std::ostream& operator<<(std::ostream& c_os,
00077                             const CCI_EyeBotLightSensor::SReading& s_reading) {
00078       c_os << "Value=<" << s_reading.Value
00079            << ">, Angle=<" << s_reading.Angle << ">";
00080       return c_os;
00081    }
00082 
00083    /****************************************/
00084    /****************************************/
00085 
00086    std::ostream& operator<<(std::ostream& c_os,
00087                             const CCI_EyeBotLightSensor::TReadings& t_readings) {
00088       if(! t_readings.empty()) {
00089          c_os << "{ " << t_readings[0].Value << " }";
00090          for(UInt32 i = 1; i < t_readings.size(); ++i) {
00091             c_os << " { " << t_readings[0].Value << " }";
00092          }
00093          c_os << std::endl;
00094       }
00095       return c_os;
00096    }
00097 
00098    /****************************************/
00099    /****************************************/
00100 
00101 }
```

---

Generated on 10 Jul 2018 for ARGoS by 
 1.6.1
